# Diagnostic value of Doppler echocardiography for identifying hemodynamic significant pulmonary valve regurgitation in tetralogy of Fallot: comparison with cardiac MRI

**DOI:** 10.1007/s10554-017-1165-4

**Published:** 2017-05-31

**Authors:** Niek E. G. Beurskens, Thomas M. Gorter, Petronella G. Pieper, Elke S. Hoendermis, Beatrijs Bartelds, Tjark Ebels, Rolf M. F. Berger, Tineke P. Willems, Joost P. van Melle

**Affiliations:** 1 Department of Cardiology, University Medical Center Groningen, University of Groningen, Hanzeplein 1, P.O. Box 30.001, 9700 RB Groningen, The Netherlands; 2 Department of Pediatric Cardiology, University Medical Center Groningen, University of Groningen, Groningen, The Netherlands; 3 Department of Cardiothoracic Surgery, University Medical Center Groningen, University of Groningen, Groningen, The Netherlands; 4 Department of Radiology, University Medical Center Groningen, University of Groningen, Groningen, The Netherlands

**Keywords:** Tetralogy of Fallot, Pulmonary regurgitation, Doppler echocardiography, Cardiac MRI

## Abstract

Quantification of pulmonary regurgitation (PR) is essential in the management of patients with repaired tetralogy of Fallot (TOF). We sought to evaluate the accuracy of first-line Doppler echocardiography in comparison with cardiac magnetic resonance imaging (MRI) to identify hemodynamic significant PR. Paired cardiac MRI and echocardiographic studies (n = 97) in patients with repaired TOF were retrospectively analyzed. Pressure half time (PHT) and pulmonary regurgitation index (PRi) were measured using continuous wave Doppler. The ratio of the color flow Doppler regurgitation jet width to pulmonary valve (PV) annulus (jet/annulus ratio) and diastolic to systolic time velocity integral (DSTVI; pulsed wave Doppler) were assessed. Accuracy of echocardiographic measurements was tested to identify significant PR as determined by phase-contrast MRI (PR fraction [PRF] ≥ 20%). Mean PRF was 29.4 ± 15.7%. PHT < 100 ms had a sensitivity of 93%, specificity 75%, positive predictive value (PPV) 92% and negative predictive value (NPV) 78% for identifying significant PR (C-statistic 0.82). PRi < 0.77 had sensitivity and specificity of 66% and 54%, respectively (C-statistic 0.63). Jet/annulus ratio ≥1/3 had sensitivity 96%, specificity 75%, PPV 92% and NPV 82% (C-statistic 0.87). DSTVI had sensitivity 84%, specificity 33%, PPV 84% and NPV 40%, (C-statistic 0.56). Combined jet/annulus ratio ≥1/3 and PHT < 100 ms was highly accurate in identifying PRF ≥ 20%, with sensitivity 97% and specificity 100%. PHT and jet/annulus ratio on Doppler echocardiography, especially when combined, are highly accurate in identifying significant PR and therefore seem useful in the follow-up of patients with repaired TOF.

**Electronic supplementary material:**

The online version of this article (doi:10.1007/s10554-017-1165-4) contains supplementary material, which is available to authorized users.

## Introduction

Chronic pulmonary regurgitation (PR) is an important sequela in patients with repaired tetralogy of Fallot (TOF), resulting in right ventricular (RV) dysfunction and dilatation, reduced exercise capacity and ventricular arrhythmias [1–4]. Pulmonary valve replacement (PVR) is the preferred treatment to improve functional outcome and to prevent further decline in RV function [5–7]. The optimal timing of PVR is however challenging and one prerequisite in the decision to perform PVR is a reliable assessment of PR severity.

Phase contrast magnetic resonance imaging (MRI) is currently considered the gold standard for the assessment of PR severity [8–10]. However, since cardiac MRI has several limitations (e.g. relatively expensive, time consuming, operator dependent and contra-indicated in patients with claustrophobia and/or implanted cardiac devices), echocardiography is the first choice in the routine follow-up of TOF patients [11]. Echocardiography could be used to identify patients in whom subsequent cardiac MRI assessment for true quantitative analysis of PR and evaluation of ventricular morphology and function is indicated. Identification of reliable echocardiographic derived parameters to identify significant PR could therefore be beneficial in economic and clinical perspective since it might help reduce expensive and unnecessary cardiac MRI procedures. For this purpose, several previously published Doppler measurements are used to estimate PR severity. However, the predictive value and accuracy of these previous suggested measurements showed contradictory results when compared to cardiac MRI [12–19]. In addition, most studies were limited by small study populations. Furthermore, the optimal threshold of the measurements for the prediction of significant PR is not uniformly defined [20].

We therefore aimed to evaluate the accuracy of Doppler echocardiography for the identification of hemodynamic significant PR in comparison with phase contrast MRI in patients with repaired TOF.

## Materials and methods

Children and adults with repaired TOF, who underwent cardiac MRI between January 2007 and March 2013, were included in this retrospective study. Patients were included if echocardiographic quality was sufficient for the assessment of all proposed echocardiographic indices. Patients were excluded when echocardiography and MRI were not performed within 3 months of each other. Also, phase contrast MRI studies with velocity aliasing or artifacts were excluded. Patients with mechanical pulmonary valve (PV) implantation prior to PR assessment were excluded as well. Baseline characteristics that were obtained included age, sex, body surface area (BSA) and surgical history.

This study complies with the Declaration of Helsinki. The local Medical Ethical Review Board of University Medical Center Groningen had no objections to the use and publication of the retrospective data. Because of the retrospective character of the study, the need for individual informed consent was waived. All assessments used in the current study were performed in the setting of regular care in these patients.

### Cardiac magnetic resonance imaging protocol

MRI protocols and image acquisitions used at our center for the assessment of pulmonary flow and ventricular volumes and function were previously described in detail [21, 22]. Briefly, MRI studies were performed on a 1.5-T scanner (Siemens, Erlangen, Germany). Two-dimensional velocity encoded MRI flow measurements, perpendicular and cranial to the pulmonary valve, were performed using 2-D gradient echo Fast Low Angle SHot, acquired during normal respiration with retrospective cardiac gating. In addition, ECG-gated cine, steady-state free precession images were acquired with breath-holding and with retrospective gating in contiguous short-axis slices covering both ventricles from base to apex.

Analyses of pulmonary flow were performed by a single experienced observer (T.M.G.) according to current recommendations [11], using QFlow 5.6 (Medis, Leiden, The Netherlands). The observer was blinded for clinical and echocardiographic variables. Pulmonary artery contours were generated semi-automatically on the standard magnitude images and were manually adjusted for each phase image. Post-processing automated background phase-offset correction is an integral part of QFlow 5.6 and was performed for each case based on previous experience [22].

Both pulmonary forward and regurgitation volume were measured. PR fraction (PRF) was calculated as regurgitation volume divided by forward volume and multiplied by 100%, and was graded as mild (<20%) or significant (≥20%) [18, 20].

Using available software (QMass 7.6, MassK, Medis, Leiden, The Netherlands), the endo- and epicardial contours of both ventricles were manually traced on the short-axis end-diastolic and end-systolic phases. For the RV, trabeculae and papillary muscle were included in the myocardial mass and excluded from the blood volume using semi-automatic threshold-based segmentation. End-diastolic, end-systolic and stroke volume, as well as ejection fraction and mass were automatically calculated using the summation of slices multiplied by slice thickness method. All absolute volumetric measurements were indexed for body surface area using Haycock’s formula [23].

### Echocardiographic protocol

Echocardiographic image acquisition was performed using a VIVID 7 echocardiographic system (General Electric, Horton, Norway) with a 2.5- to 3.5-mHz probe. Images were digitally stored for offline analysis by a single observer (N.E.G.B) using available software (GE EchoPAC version BT12). All measurements were reviewed by another experienced observer (J.P.M.). The observers were blinded for clinical and MRI variables.

**Fig. 1:**
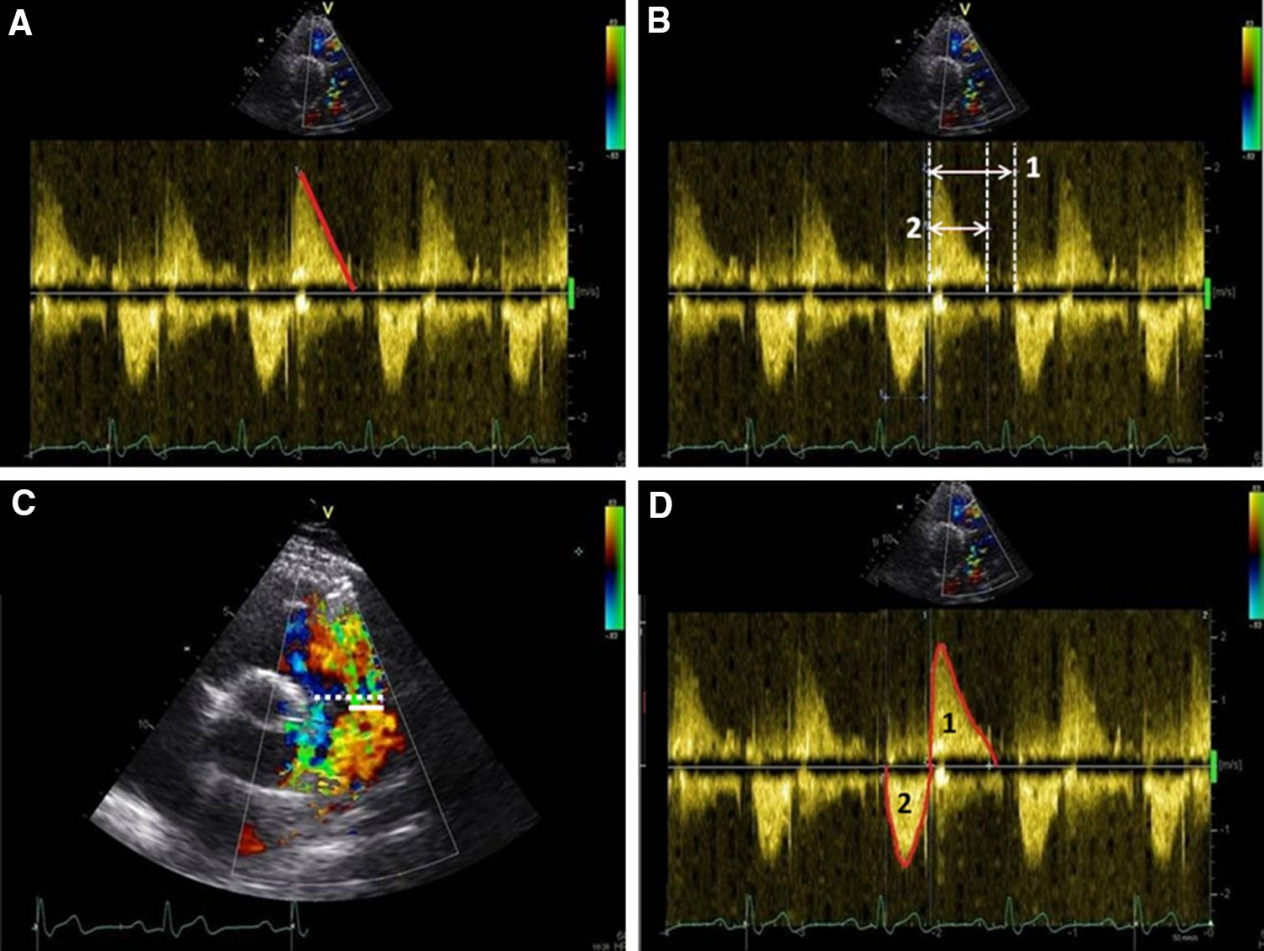
Doppler echocardiographic parameters. **a** PHT derived from CW Doppler echocardiography tracings. The deceleration slope of the PR (*red line*) represents the PHT. The PHT in this example is 118 ms, which suggests mild PR. **b** PRi measured with CW Doppler echocardiography. PRi is defined as the ratio of the duration of the regurgitation flow represented by *line 2* (in this example 433 ms) divided by the total diastolic time, *line 1* (in this example 646 ms). **c** The ratio of regurgitation jet width at the level of the PV (*straight line*) to PV annulus width (*dashed line*) measured during early diastole obtained by color-flow Doppler echocardiography. **d** DSTVI measured from the main pulmonary artery PW Doppler spectral tracing (*red lines*), is the area surface of the diastolic regurgitation flow (i.e. surface area 2) divided by the area surface of the systolic flow (i.e. surface area 1)

The pressure half time (PHT) (i.e. the time needed for the maximum transvalvular pressure gradient to decrease to its half value or the time interval for the peak velocity to reach 0.707 of the peak velocity value, in milliseconds) was measured by drawing a line along the regurgitation flow signal of the continuous wave (CW) Doppler tracing and by calculating the slope of this line according to current recommendations of the European Association of Echocardiography, as seen in Fig. 1a [24]. Previous studies have suggested PHT < 100 ms as the cut-off for significant PR [17, 18].

PR index (PRi) was calculated using the regurgitation time as percentage of the total diastolic time, derived from the CW Doppler tracings. The total diastolic time was defined as the time from the beginning of the diastolic flow curve to the beginning of the next systolic flow curve. The PR time was calculated from the beginning of the diastolic flow curve to the equilibration point at the x-axis, as seen in Fig. 1b. A low PRi corresponds with more severe regurgitation. Li et al. and Renella et al. have suggested PRi < 0.77 as the cut-off value between mild and significant PR [14, 17].

The jet diameter was measured during early diastole using Color Doppler while avoiding aliasing by adjusting the Nyquist limit (Fig. 1c). The annulus diameter was measured in the same view using 2D images without color. Both measurements were used to calculate the jet/annulus ratio [24]. Regarding jet/annulus ratio, the optimal threshold for identifying significant PR has been reported, but inconsistently [13–17].

The pulsed-wave (PW) Doppler tracing from the main pulmonary artery was used to obtain the velocity–time integrals of diastolic and systolic pulsed-waves (Fig. 1d). The diastolic to systolic time velocity integral (DSTVI) was calculated by dividing the surface area of the diastolic flow curve by the surface area of the systolic flow curve. Mercer-Rosa et al. defined a DSTVI > 0.49 as the cut-off between mild and hemodynamically significant PR [15]. The optimal threshold for the identification of significant PR for all 4 Doppler echocardiographic measurements were re-assessed.

Inter-observer and intra-observer variability for PHT, PRi and jet/annulus ratio was assessed using 20 randomly selected echocardiographic studies and was performed by 2 independent observers (N.E.G.B. and T.M.G.), who were blinded for the clinical, MRI and echocardiographic variables. The interval between the intra-observer analyses was more than 6 months.

### Statistical analysis

Values are expressed as mean ± standard deviation for normal distributed data, median (interquartile range) for skewed distributed variables or as number (percentage) for categorical data. The sensitivity, specificity, positive predictive value (PPV) and negative predictive value (NPV) for the identification of PRF ≥ 20% were calculated for each echocardiographic measurement. The Pearson r correlation coefficient was used to determine the correlation between continuous variables. Comparisons between groups were performed using independent samples T-test. The most optimal cut-off point for PHT, PRi, jet/ annulus ratio and DSTVI for identifying significant PR was determined by plotting a receiver operating characteristic (ROC) curve. Accuracy of each test was determined by calculating the C-statistic. For inter- and intra-observer variability, the Two-way mixed Intraclass Correlation Coefficient was used. Statistical significance was considered achieved at a p value <0.05. Statistical analyses were performed using SPSS statistical software (Version 22, 2013).

## Results

**Fig. 2:**
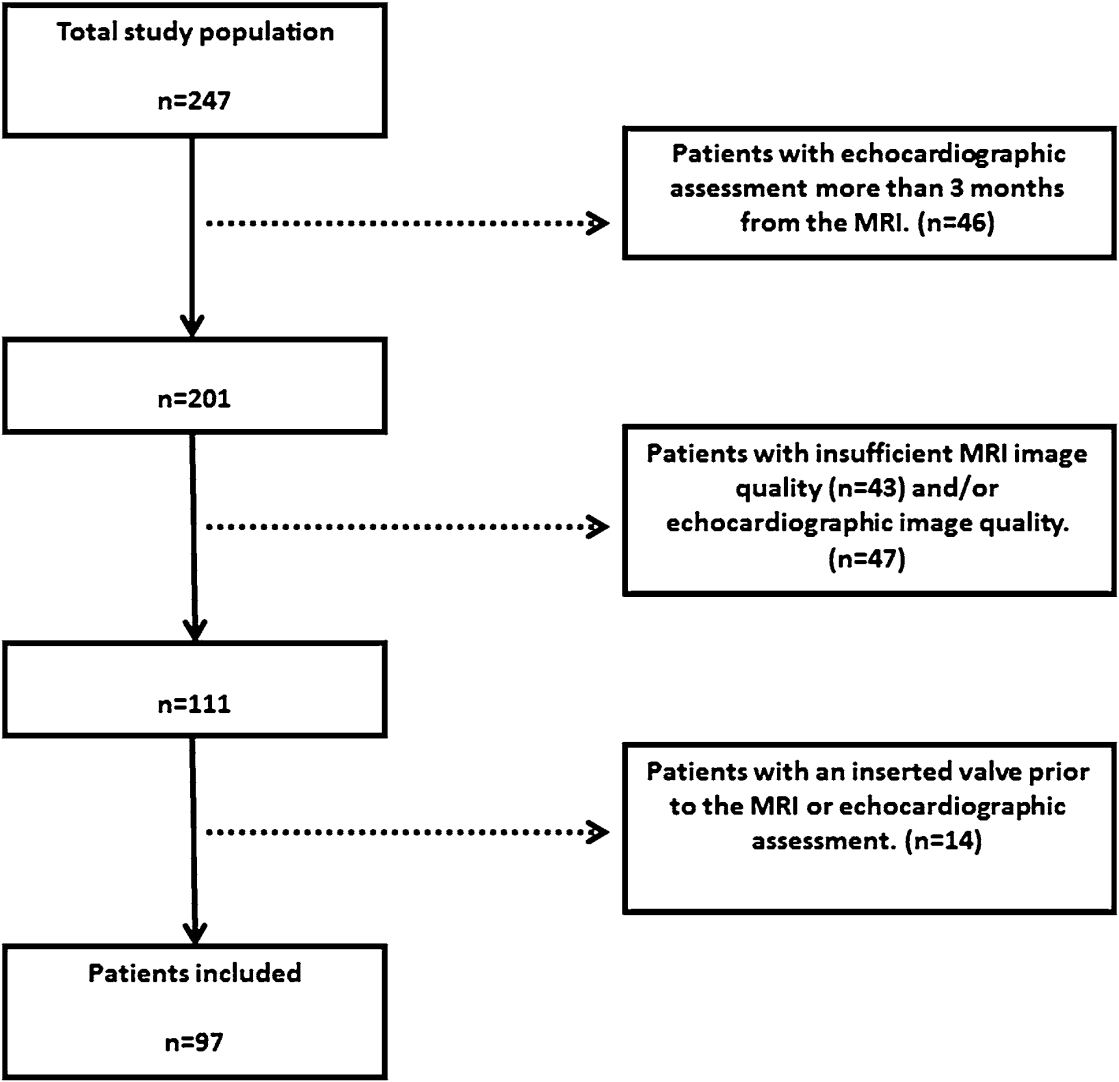
Flow chart of the study population

Figure 2 demonstrates the flow chart of the study population. Table 1 summarizes the baseline characteristics of the total study population. In total, 97 patients (50.5% male) with repaired TOF were included in the study. The mean age of the study population was 28.4 ± 11.5 years. The median interval between the echocardiographic assessment and the phase contrast MRI was 35 (IQR 16–56) days. Right ventricular volumetric and functional parameters are illustrated in Table 1. As suspected, PRF correlated with RVEDVi (0.64, p < 0.001) and RVESVi (0.50, p < 0.001). Of the total study cohort, 24 patients had mild PR (25%) and 73 had significant PR (75%).

**Table 1:** Baseline characteristics of the total study population (n = 97)

Demographics	
Sex (male)	49 (50.5%)
Age (years)	28.4 ± 11.5
BSA (m^2^)	1.81 ± 0.3
Surgical history	
Type of initial correction	
TAP	60 (62%)
No-TAP	32 (33%)
Conduit	2 (2%)
Unknown	3 (3%)
Age at initial correction (years)	3.15 ± 3.62
MRI	
Time from initial correction to MRI (years)	25.3 ± 8.87
RVEDVi (ml/m^2^)	132 ± 36.3
RVEF (%)	49.7 ± 6.9
PRV(ml/m^2^)	20.2 ± 15.3
PRF (%)	29.4 ± 15.7
Echocardiography	
PHT (ms)	86.7 ± 29.6
PRi	0.72 ± 0.15
Jet/annulus ratio	0.52 ± 0.19
DSTVI	0.88 ± 0.38
TAPSE (mm)	18.9 ± 3.78
PV peak gradient (mmHg)	22.2 ± 12.3

Variables are expressed as n (%), mean ± SD or median (interquartile range)

*BSA* body surface area, *DSTVI* diastolic to systolic time velocity integral, *PHT* pressure half time, *PRF* pulmonary regurgitation fraction, *PRi* pulmonary regurgitation index, *PRV* pulmonary regurgitation volume, *PV* pulmonary valve, *RVEDVi* right ventricular end diastolic volume index, *RVEF* right ventricular ejection fraction, *TAP* transannular patch, *TAPSE* tricuspid annular plane systolic excursion

**Fig. 3:**
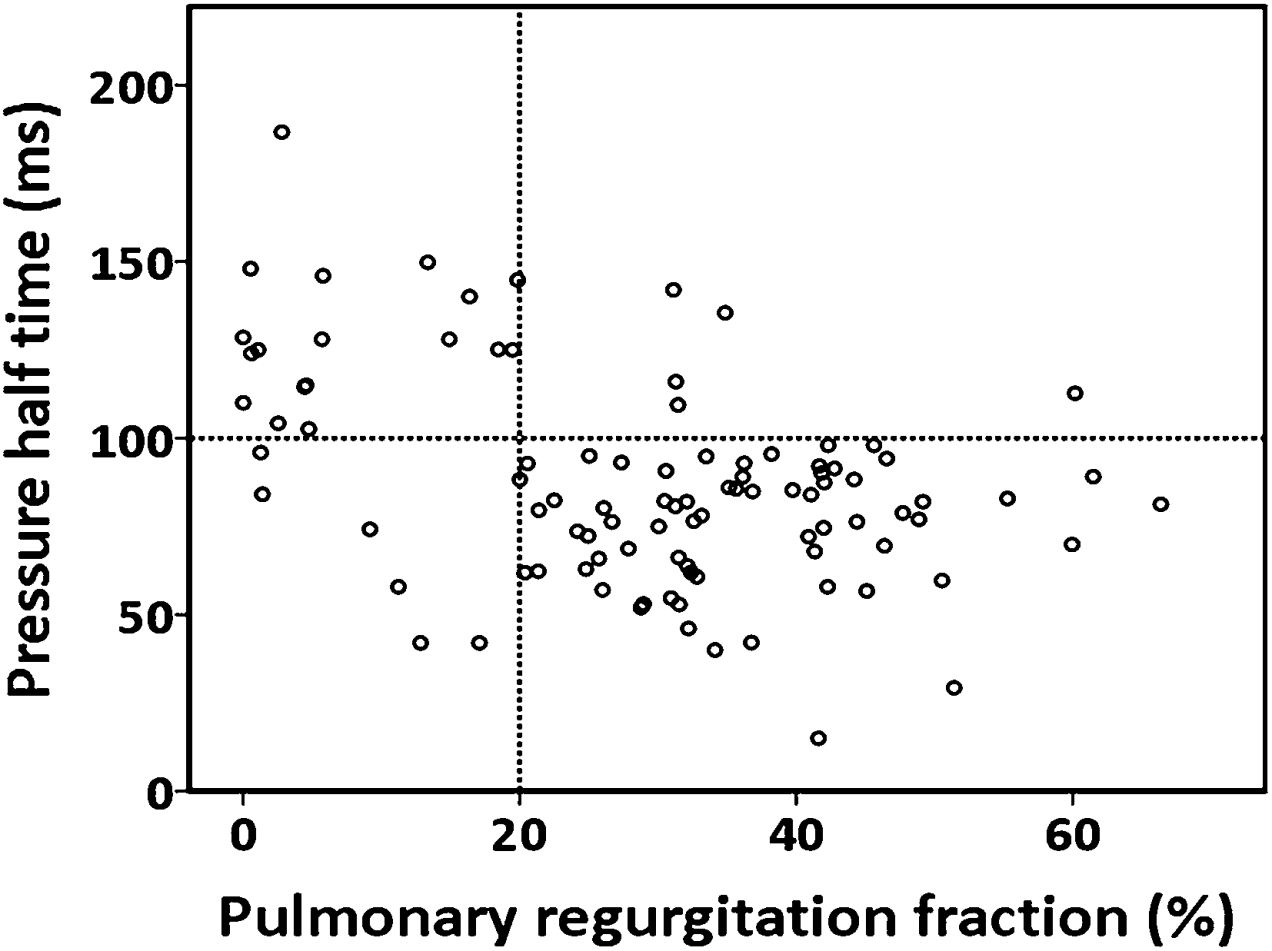
Relationship between PHT measured by CW Doppler echocardiography and PRF from MRI. The* dotted vertical line* marks the transition from insignificant to significant PR by MRI (i.e. PRF 20%). The* dotted horizontal line* corresponds with the echocardiographic cut-off point for significant PR (i.e. PHT 100 ms)

**Fig. 4:**
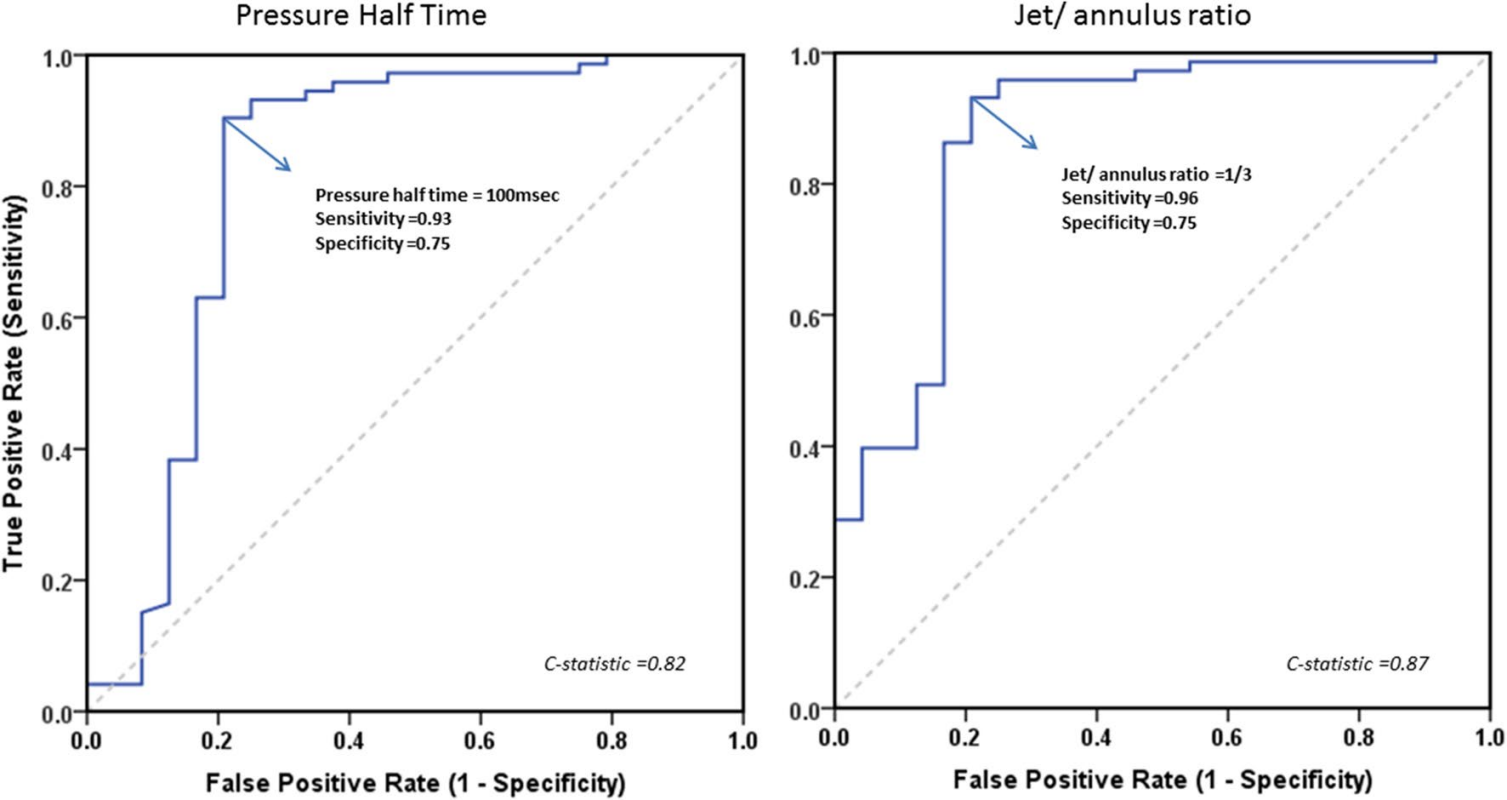
ROC curve ratio representing the PHT and jet/annulus ratio measured by Doppler echocardiography for identifying PR ≥ 20% on MRI. The accuracy of the tests are measured by the area under the curve (AUC). In this figure the AUC for PHT was 0.82 and for jet/annulus ratio 0.87. The PHT cut-off 100 ms and a jet/annulus ratio cut-off of 1/3 corresponds is reliable in separating mild from significant pulmonary regurgitation

**Fig. 5:**
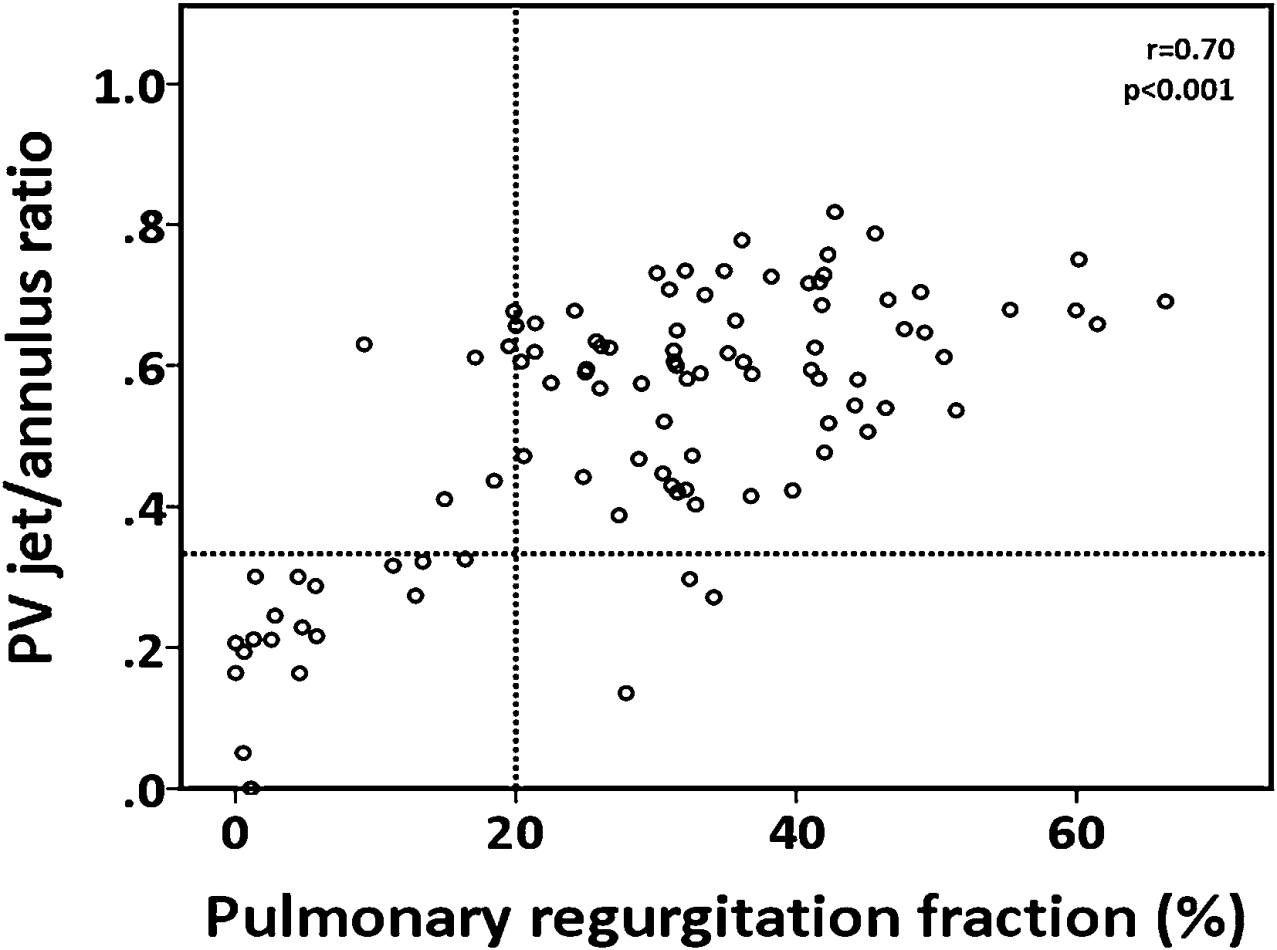
Scatter plot of the jet/annulus ratio and phase-contrast MRI-derived PRF. The *dotted vertical line* marks the transition from insignificant to significant PR by MRI (i.e. PRF 20%). The *dotted horizontal line* is the optimal cut-off value of significant PR (i.e. jet/annulus ratio 1/3)

A detailed overview of the predictive value of all echocardiographic parameters for identifying significant PR is illustrated in Table 2. The relationship between PHT and PRF is shown in Fig. 3. For the identification of PRF ≥ 20%, the C-statistic for PHT in the ROC analysis was 0.82 (p < 0.001) with an optimal cut-off value of <100 ms (Fig. 4). Using this cut-off, PHT demonstrated good sensitivity, specificity, positive predictive value (PPV), and negative predictive value (NPV) for identifying significant PR. (Table 2) For PRi, the C-statistic in the ROC analysis was 0.63 (p < 0.001). No optimal threshold for PRi could be determined to identify significant PR. In the current study, the previous suggested PRi threshold of <0.77 had a poor sensitivity, specificity and NPV for identifying patients with significant PR. In contrast, the PPV was acceptable. There was no significant correlation between heart rate and PRi (r = 0.172, p = 0.093). Jet/annulus ratio was strongly correlated with PRF (r = 0.70, p < 0.001). (Fig. 5). The C-statistic was 0.87 (p < 0.001), with an optimal jet/annulus ratio cut-off of 1/3 (Fig. 4). The jet/annulus ratio ≥1/3 demonstrated strong sensitivity, specificity, PPV and NPV to identify significant PR. Using this cut-off, a significant difference in PRF between groups was seen, as shown in the Supplementary data. DSTVI was poorly correlated with PRF (r = 0.18, p = 0.08). The C-statistic was 0.56 with an optimal DSTVI cut-off of 0.61, to differentiate between mild and significant PR. DSTVI < 0.61 for identifying significant PR showed good sensitivity and PPV, however it had very poor specificity and NPV.

**Table 2:** Sensitivity and specificity of echocardiographic parameters in identifying significant pulmonary regurgitation

	PRF on MRI (%)		
	(≥20)	(<20)	Total
**Pressure half time**			
* Sensitivity: 93, specificity: 75* *PPV: 92, NPV: 78*			
Significant (<100 ms)	68	6	74
Mild (≥100 ms)	5	18	23
Total	73	24	97
**Pulmonary regurgitation index**			
* Sensitivity: 66, specificity: 54* *PPV: 81, NPV: 34*			
Significant (<0.77)	48	11	59
Mild (≥0.77)	25	13	38
Total	73	24	97
**PV jet/ annulus ratio**			
* Sensitivity: 96, specificity 75* *PPV: 92, NPV: 86*			
Significant (≥1/3)	70	6	76
Mild (<1/3)	3	18	21
Total	73	24	97
**DSTVI**			
* Sensitivity: 84, specificity: 33* * PPV: 79, NPV: 40*			
Significant (≥0.61)	61	16	77
Mild (<0.61)	12	8	20
Total	73	24	97

*DSTVI* diastolic to systolic time velocity integral, *NPV* negative predictive value,* PPV* positive predictive value,* PRF* pulmonary regurgitation fraction,* PV* pulmonary valve

Since jet/annulus ratio and PHT appeared to be the most sensitive in identifying significant PR, we tested the combination of these 2 measurements to form the following groups: patients in whom both Doppler variables were in the non-significant range (i.e. PHT > 100 ms and jet/annulus ratio <1/3: group 1), patients in whom only 1 of above mentioned Doppler variables was in the significant range (i.e. PHT < 100 ms or jet/annulus ratio ≥1/3: group 2), and finally patients in whom both Doppler variables were in the significant range (i.e. PHT < 100 ms and jet/annulus ratio ≥1/3: group 3). The results can be seen in Table 3. The combination of jet/annulus ratio ≥1/3 and PHT < 100 ms was highly accurate in identifying significant PR (i.e. sensitivity 97%, specificity 100%).

**Table 3:** Combination of echocardiographic derived jet/annulus ratio and PHT compared to PRF on MRI

	Group 1	Group 2	Group 3	Total
PRF < 20%	14	8	2	24
PRF ≥ 20%	0	8	65	73
Total	14	16	67	97

*Group 1* jet/annulus < 1/3 and PHT ≥ 100 ms; *group 2:* jet/annulus ratio ≥1/3 and PHT > 100 ms OR jet/annulus ratio < 1/3 and PHT < 100 ms; and *group 3* jet/annulus ≥ 1/3 and PHT < 100 ms

*PRF* pulmonary regurgitation fraction

The interclass correlation for the inter-observer variability for PHT, PRi and jet/annulus ratio was 0.89 (95% CI 0.47–0.94, p < 0.001), 0.80 (95% CI 0.31–0.94, p < 0.006) and 0.87 (95% CI 0.54–0.96, p < 0.001), respectively. For the intra-observer measurements, the correlation for PHT was 0.98 (95% CI 0.92–0.99, p < 0.001), for PRi 0.80 (95% CI 0.30–0.94, p < 0.007) and for jet/annulus ratio the correlation was 0.93 (95% CI 0.76–0.98, p < 0.001).

## Discussion

This study demonstrates that the easily obtainable transthoracic echocardiographic measurements PHT and jet/annulus ratio are accurate in identifying significant PR, assessed with MRI. In contrast, CW Doppler-derived PR index and DSTVI appeared to be inferior measurements in distinguishing significant from non-significant PR. The proposed echo-parameters could be used as a diagnostic tool to identify patients who should undergo subsequent cardiac MRI assessment for the evaluation of PR severity and ventricular morphology and function. This could be interesting from a clinical and economic perspective as it might help reduce unnecessary and costly diagnostic procedures such as cardiac MRI.

Previous studies investigated PHT in comparison with phase contrast MRI, and suggested that PHT < 100 ms was both sensitive and specific in identifying significant PR compared with MRI-determined PRF ≥ 20% [17, 18]. However, the number of patients included in these studies was rather small (n = 26 and n = 34, respectively). In the current study, PHT < 100 ms proved to be reliable in identifying significant PR. However, PHT depends not only on PR severity, but also on factors that may affect the equilibration of the transvalvular pressures (e.g. diastolic intrapulmonary pressures, diastolic properties of the RV and anatomical obstructions in the right ventricular outflow tract) [24].

Both Li et al. and Renella et al. have previously investigated the value of PRi on CW Doppler echocardiography to identify significant PR [14, 17]. The current results are in line with Renella et al. in that PRi demonstrated to be unreliable in identifying significant PR. This might partially be explained by the fact that PRi measurements are also highly dependent on dynamic pressure gradients, which may lead to early equilibration of the transvalvular pressures. The shorter duration of PR in, for instance, a restrictive physiology might be associated with low PRi (especially at a low heart rate) and may overestimate the severity of PR [17, 25]. Of note, we observed no significant correlation between heart rate and PRi.

In our study, the accuracy of the jet/annulus ratio was reliable in distinguishing mild from significant PR. The optimal thresholds for mild and significant PR regarding the jet/annulus ratio has been reported, but inconsistently [13–17]. Puchalski et al. and Renella et al. demonstrated a jet/ annulus ratio of >0.5 predictive of PRF ≥ 20%, whereas Mercer-Rosa et al. demostrated a jet/ annulus of 0.25 accurate in identifying PRF > 20% [15–17]. Yet in the present study, we observed that a jet/annulus ratio of ≥1/3 may be a potential reliable cutoff for significant PR, which is in line with the findings of Grothoff et al. [13] Of note, the jet may not fully be represented in a single echocardiographic plane. Furthermore, disruption of the pulmonary valve in patients with TOF can results in distortion of the RVOT particularly those after transannular patch and therefore makes accurate standardized measurement of the PV annulus difficult. Nevertheless, despite the aforementioned limitations the jet/ annulus ratio was most accurate in identifying significant PR.

Time velocity integral is used for the calculation of cardiac output and the severity of aortic regurgitation, but little is known about its value for assessing PR. Mercer-Rosa and co-workers concluded that DSTVI can be helpful for identifying significant PR, given its moderate correlation with MRI derived PRF, as well as its high sensitivity and specificity [15]. In contrast, in our study the correlation between DSTVI and PRF was rather weak. There is a difference between Mercer-Rosa et al. and the present study that merit emphasis. In Mercer-Rosa et al. study, a DSTVI of 0.49 corresponded to MRI PRF of 20% and was used as a cut-off for identifying significant PR, whereas in this study a echocardiographic derived DSTVI cut-off value of 1/3 was used to distinguish between significant and non-significant PR.

To our knowledge, we are the first to describe the combination of PHT and jet/ annulus to identify significant PR. The probability significant PR will be detected by Doppler echocardiography is 97%, using PHT < 100 ms and jet/ annulus ratio <1/3. Remarkably, the specificity of the combined indices in estimating significant PRF was 100%. From a practical perspective, these findings could function as an additional diagnostic tool in the follow up of patients with repaired TOF. Yet, CMR remains the cornerstone in the decision-making process of revalvulation of the RVOT since it provides precise evaluation of ventricular morphology and function.

Regarding the definition of hemodynamic significant PR on MRI, the optimal PRF cut-off value is subject of debate [12–20]. Published studies used PRF 20, 24.5 and 40% as a cut-off on MRI for identifying significant or severe PR [12–20]. In patients with normal pulmonary artery pressures, PRF ≥ 40% is relatively uncommon due to fast equilibration of pulmonary artery and diastolic RV pressures [20]. In addition, Silversides et al. concluded that there was no significant difference in magnitude of RV enlargement between PRF 20 and 40% [18]. Therefore, in line with previous published studies, a PRF ≥ 20% as the cut-off on MRI to identify significant PR was used in the current study [17, 18].

Further prospective studies could be helpful for the refinement of the proposed criteria for the grading of PR fraction. Longitudinal studies are required to establish whether these criteria could be translated in improved timing of pulmonary valve replacement and outcome of repaired TOF patients. Moreover, prospective analysis is recommended for the independent confirmation of the ROC threshold values identified in the current analyses. Additionally, prospective image collection focused on previous described parameters is recommended for obtaining a higher percentage of subjects with sufficient echocardiographic and MRI quality.

### Limitations

This retrospective study is accompanied by several limitations. First, although the interval between the MRI and echocardiographic assessment was limited to a median of 35 days, the severity of PR may vary within patients at different time points related to their hemodynamic state. Second, continuous-wave Doppler indices may be influenced by other haemodynamic parameters such as pulmonary pressure and RV compliance. Unfortunately, invasively derived data on RV and pulmonary artery elasticity and compliance were lacking. Third, the majority of patients in this study were adults and therefore the results may not be generalizable to the pediatric population.

## Conclusion

Doppler echocardiography for the assessment of the severity of PR is an useful tool in the follow up of TOF patients. The easily accessible transthoracic echo variables PHT and jet/annulus ratio, especially when combined, have the highest diagnostic value in identifying significant PR in comparison with the considered gold standard MRI in patients with repaired TOF.

## Electronic supplementary material

Below is the link to the electronic supplementary material.

Supplementary material 1 (DOCX 79 KB)
