# Supplementary material for: Diagnostic value of Doppler echocardiography for identifying hemodynamic significant pulmonary valve regurgitation in tetralogy of Fallot: comparison with cardiac MRI
Source: Int J Cardiovasc Imaging. 2017 May 31;33(11):1723–30. doi: 10.1007/s10554-017-1165-4 (PMC5682867; doi:10.1007/s10554-017-1165-4)

**SUPPLEMENTARY DATA**

**Figure S1:** boxplot comparing the severity of PR as determined by MRI and the jet/annulus ratio obtained by color-flow Doppler echocardiography. Pulmonary regurgitation fraction is significant higher in patients with a jet/annulus ratio ≥1/3 than in patients with jet/annulus ratio <1/3, p<0.001.


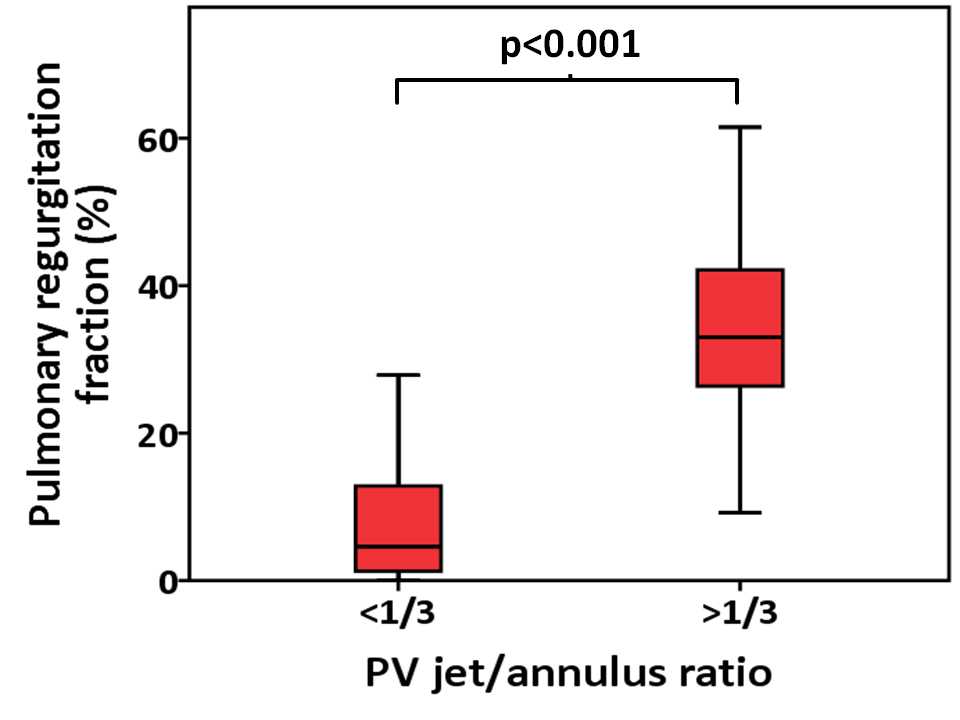

Supplement: Supplementary file 1 — Supplementary material 1 (DOCX 79 KB) [file 10554_2017_1165_MOESM1_ESM.docx]
